# The role of glial cell line‐derived neurotrophic factor family member artemin in neurological disorders and cancers

**DOI:** 10.1111/cpr.12860

**Published:** 2020-06-23

**Authors:** Sipin Zhu, Yihe Li, Samuel Bennett, Junhao Chen, Isabel Ziwai Weng, Lin Huang, Huazi Xu, Jiake Xu

**Affiliations:** ^1^ Department of Orthopaedics The Second Affiliated Hospital and Yuying Children's Hospital of Wenzhou Medical University Wenzhou China; ^2^ School of Biomedical Sciences University of Western Australia Perth WA Australia; ^3^ Department of Spine Surgery Sun Yat‐sen Memorial Hospital Sun Yat‐sen University Guangzhou China; ^4^ Department of Orthopaedics Sun Yat‐sen Memorial Hospital Sun Yat‐sen University Guangzhou China

**Keywords:** artemin, cancers, glial cell line‐derived neurotrophic factor, neurological disorders

## Abstract

Artemin (ARTN) is a member of the glial cell line‐derived neurotrophic factor (GDNF) family ligands (GFLs), which encompasses family members, GDNF, neurturin (NRTN) and persephin (PSPN). ARTN is also referred to as Enovin or Neublastin, and bears structural characteristics of the TGF‐β superfamily. ARTN contains a dibasic cleavage site (RXXR) that is predicted to be cleaved by furin to yield a carboxy‐terminal 113 amino acid mature form. ARTN binds preferentially to receptor GFRα3, coupled to a receptor tyrosine kinase RET, forming a signalling complex for the regulation of intracellular pathways that affect diverse outcomes of nervous system development and homoeostasis. Standard signalling cascades activated by GFLs via RET include the phosphorylation of mitogen‐activated protein kinase or MAPK (p‐ERK, p‐p38 and p‐JNK), PI3K‐AKT and Src. Neural cell adhesion molecule (NCAM) is an alternative signalling receptor for ARTN in the presence of GFRα1, leading to activation of Fyn and FAK. Further, ARTN also interacts with heparan sulphate proteoglycan syndecan‐3 and mediates non‐RET signalling via activation of Src kinases. This review discusses the role of ARTN in spinal cord injury, neuropathic pain and other neurological disorders. Additionally, ARTN plays a role in non‐neuron tissues, such as the formation of Peyer's patch‐like structures in the lymphoid tissue of the gut. The emerging role of ARTN in cancers and therapeutic resistance to cancers is also explored. Further research is necessary to determine the function of ARTN in a tissue‐specific manner, including its signalling mechanisms, in order to improve the therapeutic potential of ARTN in human diseases.

## 1 INTRODUCTION

Glial cell line‐derived neurotrophic factor (GDNF) was identified by its ability to support the survival of midbrain dopaminergic neurons, and as a distant relative of the transforming growth factor‐beta (TGF‐β) superfamily.^1^ The GDNF family ligands (GFLs), including GDNF, neurturin (NRTN), artemin (ARTN) and persephin (PSPN), are vital for the development and maintenance of homoeostasis of the central and peripheral neurons of the mammalian nervous system.^2^, ^3^ ARTN is also known as Enovin^4^ or Neublastin,^5^ and exhibits structural characteristics as a dimeric and cysteine‐knot motif like molecule, which resembles distant members of the TGF‐β superfamily.^2^, ^6^, ^7^, ^8^

Under physiological conditions, ARTN promotes sensory neuron survival and peripheral nerve homoeostasis,^9^ and the cell survival of dopaminergic neurons of the ventral mesencephalon in the brain.^10^ ARTN knockout mice revealed abnormalities in the sympathetic nervous system (SNS), with defective migration and axonal projection pattern of SNS.^11^ In pathological nerve injury, ARTN plays a distinct role in neuropathic pain and morphological alterations of nerves.^12^ In addition to its role in neural tissue tropism, ARTN acts as an attractant of intestinal hematopoietic cells and participates in the formation of Peyer's patch‐like structures in the gut.^4^, ^9^

This review discusses the role of ARTN in spinal cord injury repair, neuropathic pain and other neurological disorders. An overview of the molecular structure, signalling pathways, and gene expression of ARTN is presented. In addition, the emerging role of ARTN in various types of cancers is surveyed. Further understanding of the role of ARTN in a tissue‐specific manner and its underlying signalling mechanisms will help us to develop ARTN as a therapeutic target for neurological diseases and cancers.

## 2 MOLECULAR STRUCTURE AND EXPRESSION OF ARTN

**FIGURE 1:**
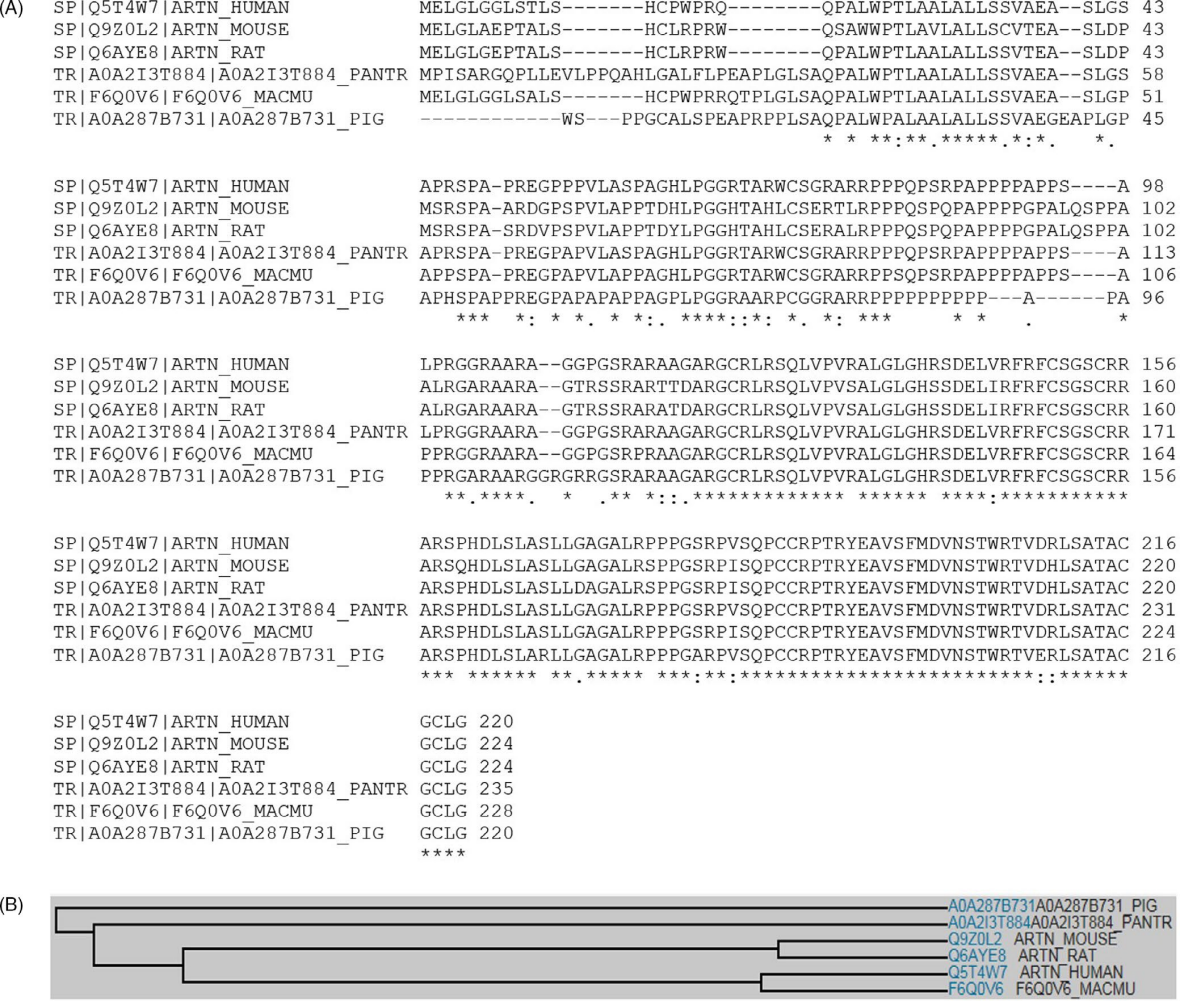
ARTN amino acid sequence analysis. A, Multiple sequence analysis reveals that human ARTN shares considerable amino acid sequence identity to rat, mouse, chimpanzee, rhesus macaque and pig ARTN homologs, particularly in the mature form at the C terminus, indicating that ARTN is a well‐conserved protein among mammalian species. B, Family tree of ARTN in various species

Multiple sequence analyses indicate that human ARTN shares considerable (approximately 60%‐65%) amino acid sequence identity to rat, mouse, pig, pantry and rhesus macaque ARTN homologs, particularly at their carboxyl termini (approximately 85%‐90%), indicating that ARTN is a well‐conserved protein among mammalian species (Figure 1A,B).

**FIGURE 2:**
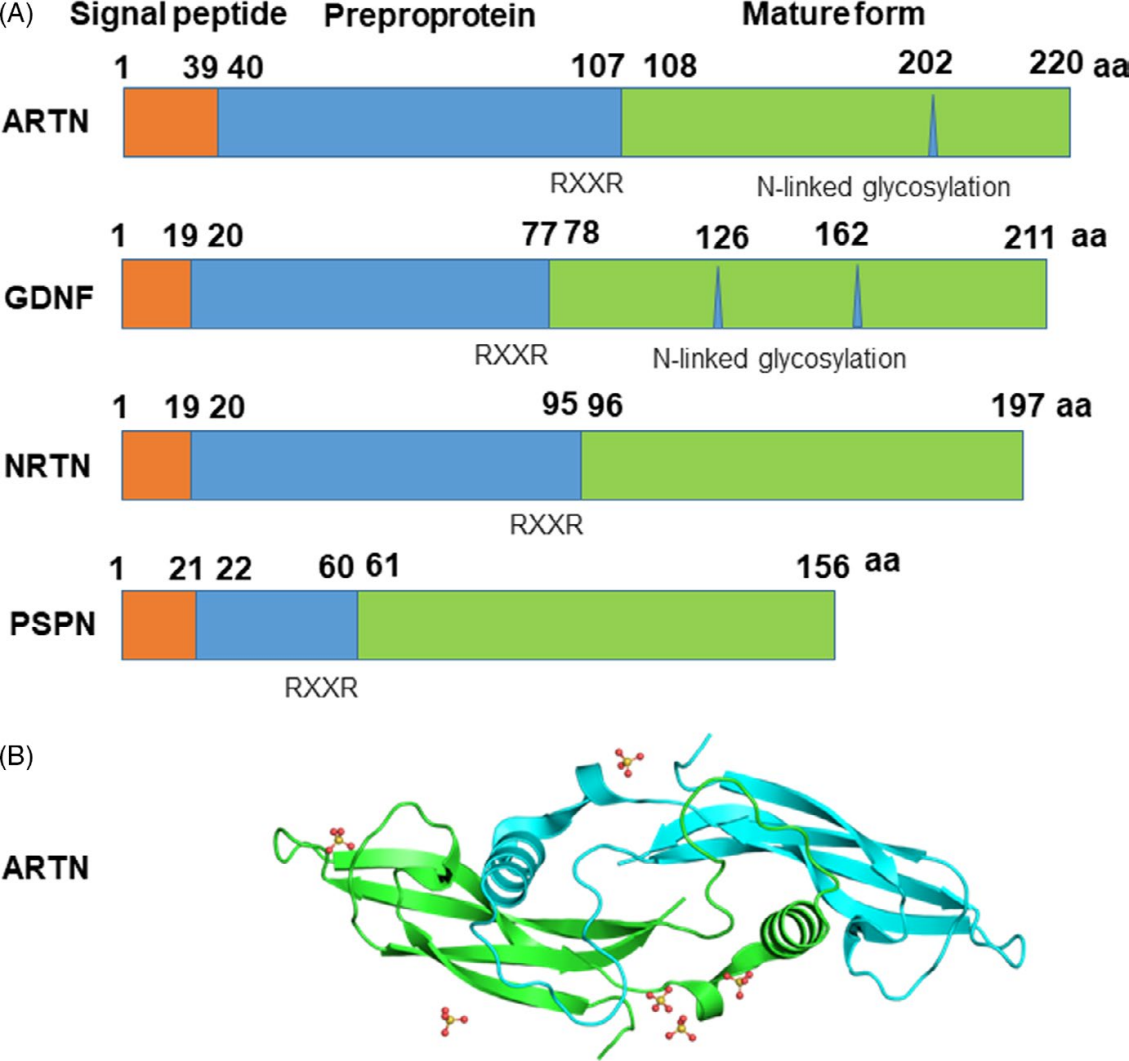
Molecular structure of ARTN. A, Human homolog of ARTN contains an N terminal signal sequence and is expressed as a preproprotein, which contains a putative furin cleavage site of RXXR at amino acid residue 107, and is further processed as a mature form of a 113 amino acid functional ARTN with a putative glycosylation site, and disulphide‐linked homodimer of 28 kDa peptide. ARTN shares a similar structure with GDNF, NRTN and PSPN which also contain putative furin cleavage site of RXXR at amino acid residues 77, 95 and 60, respectively. B, Tertiary structure analysis showing that ARTN shares typical features of TGF‐β superfamily as predicted by EBI‐based bioinformatics (https://www.ebi.ac.uk/pdbe/entry/pdb/2ASK)

At the molecular structural level, human ARTN contains an amino‐terminal signal sequence and is expressed as a preproprotein, which undergoes further protein cleavage at the dibasic motif (RXXR) predicted to be cleaved by furin to yield a carboxy‐terminal 113 amino acid mature form of functional ARTN with a disulphide‐linked homodimer of 28 kDa protein (Figure 2A).^4^, ^5^, ^9^ ARTN bears sequence similarity with GDNF, NRTN and PSPN which also contain putative furin cleavage site of RXXR at amino acid residues 77, 95 and 60, respectively (Figure 2A).^9^ Recombinant ARTN proteins were expressed and purified from bacterial hosts, and exhibited biological activity associated with disulphide structural folding.^13^, ^14^ Three dimensional structure of ARTN is known, and it resembles that of GDNF and NRTN, and has some homology with TGF‐beta family members (Figure 2B).^15^

At the transcriptional level, ARTN mRNAs were reported to be expressed in developing nerve roots,^9^ Schwann cells^6^ and embryonic vascular smooth muscle cells.^11^ The expression of ARTN was regulated by the master activator protein 1 (AP‐1) transcription factor, c‐Jun, in Schwann cells.^16^ Upregulation of ARTN was also identified in mesenchymal stem cells (MSCs) transplanted onto the cortex of brain injured rats, indicating a potential role of ARTN in central neural tissue regeneration.^17^

## 3 ARTN RECEPTOR AND SIGNALLING PATHWAYS

**FIGURE 3:**
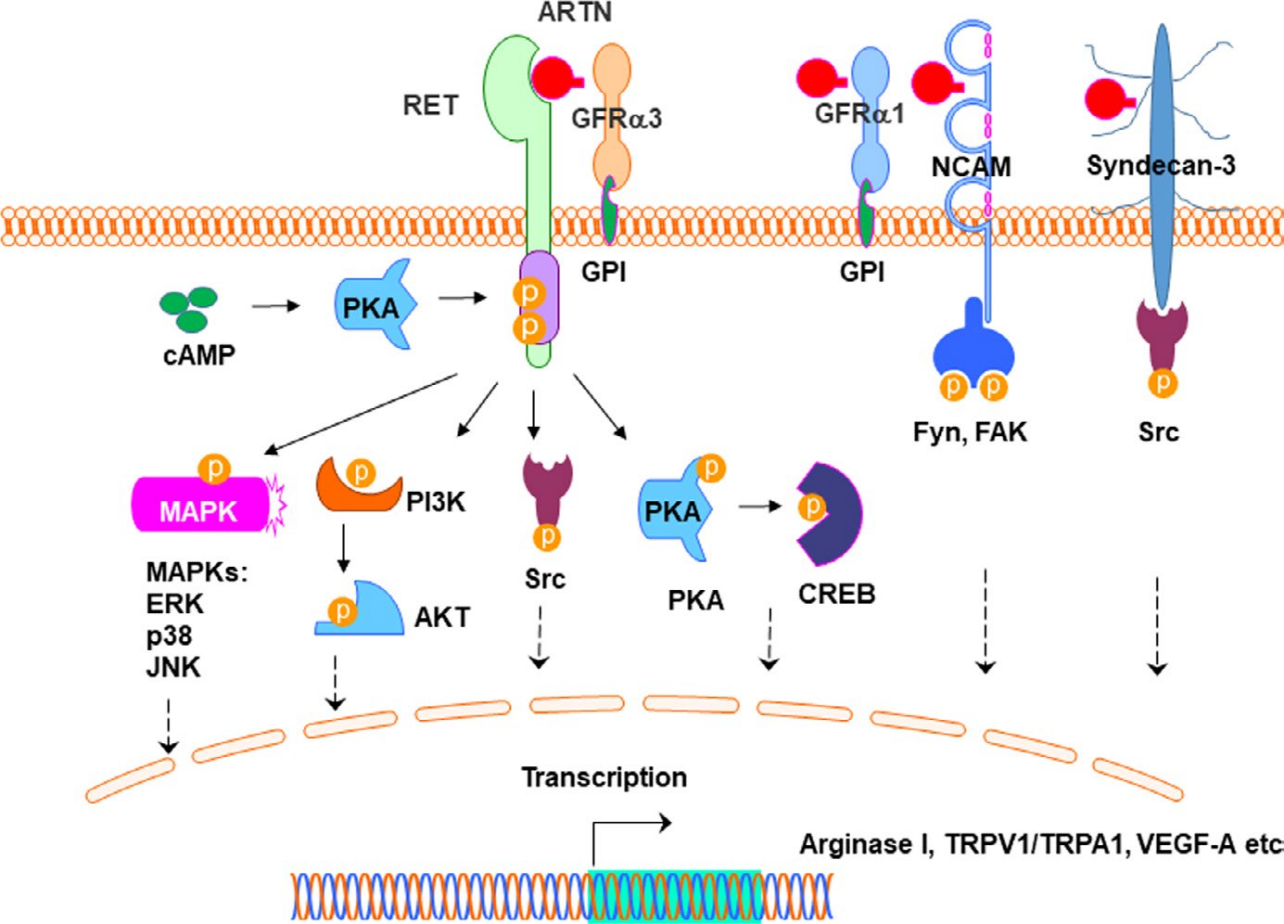
ARTN signalling. ARTN mediates the activation of the GFRα3/RET receptor complex. ARTN binds to GFRα3 which is attached to the membrane via GPI anchor. The GFRα1/RET complex has also been suggested to be a putative receptor for ARTN. Several cascades activated by GFLs via RET include the phosphorylation of MAPK (p‐ERK, p‐p38 and p‐JNK), PI3K‐AKT and Src. In addition, NCAM is an alternative signalling receptor for ARTN only in the presence of GFRα1, leading to activation of Fyn and FAK. Further, ARTN also interacts with heparan sulphate proteoglycan syndecan‐3 and mediates non‐RET signalling via activation of Src‐type kinases. Interestingly, PKA might act as regulator of upstream of RET via cAMP, or as a downstream regulator of RET

GFLs, including ARTN, signal by the formation of a complex receptor system consisting of a ligand‐specific non‐signalling receptor subunit GFRα, coupled to a receptor tyrosine kinase, RET (Figure 3).^18^, ^19^, ^20^ Four GFRα receptors have been identified (GFRα1‐4), and ARTN first binds to GFRα3 which is attached to the membrane via glycosylphosphatidylinositol (GPI) anchor.^9^, ^21^ And then the ARTN‐GFRα3 complex binds to and activates receptor tyrosine kinase RET by triggering phosphorylation of RET intracellular tyrosine residues,^21^, ^22^ and this pathway is essential for migration, axonal growth and axon guidance of developing sympathetic neurons.^23^ GFRα3 was found to be strongly expressed in dorsal root ganglia cells and Schwann cells,^24^ and in the developing brain, as well as in developing and adult peripheral nerves.^25^ Consistently, RET mRNA was strongly expressed in motor neurons and dorsal root ganglion neurons.^24^

Several cascades activated by GFLs via RET include the phosphorylation of MAPK (p‐ERK, p‐p38 and p‐JNK), PI3‐AKT and Src (Figure 3).^26^, ^27^, ^28^, ^29^ GFL signalling is diverse, and ARTN could also signal independently of RET in combination with other receptors.^2^, ^7^, ^9^, ^30^ The GFRα1 has been suggested to be a receptor for ARTN,^9^, ^31^ but displays much weaker binding to ARTN than GDNF^32^ and lack of functional role in ARTN‐mediated cell activity.^30^, ^33^, ^34^ In addition, GDNF signalling was identified via the neural cell adhesion molecule (NCAM), which is distinct from RET signalling receptor for GDNF family ligands. Interaction of NCAM with GFRα1 facilitates high‐affinity binding of ARTN to NCAM, leading to activation of non‐receptor tyrosine‐protein kinase Fyn and focal adhesion kinase (FAK) in cells lacking RET (Figure 3).^32^, ^35^ ARTN also binds to the heparin sulphate side chains of syndecan‐3 and activates Src pathways, and this signalling does not require the presence of GFRα receptors (Figure 3).^7^, ^36^

It was observed that intracellular cyclic adenosine monophosphate (cAMP) elevation leads to the activation of protein kinase A (PKA) in neuronal cells.^37^ Serine 696 in RET was identified as a putative phosphorylation site by upstream PKA, whereas phosphorylation of tyrosine 1062 in RET is crucial for the downstream activation of phosphatidylinositol 3‐kinase (PI3K).^37^ Consistently, using Ret‐null Wolffian ducts in a budding experiment, it was shown that PKA is involved in GDNF‐dependent effect.^38^ (Figure 3). On the other hand, PKA might also act as a downstream regulator of RET. Using by primary dorsal root ganglion neurons in culture, ARTN was found to support the extension of neurites through activation of PKA to phosphorylate cAMP‐response element binding protein (CREB) (Figure 3).^39^

The utility of ARTN signalling is exemplified by regulating the expression of genes involved in various biological processes, including arginase I in spinal cord neurons,^39^ TRPV1 in cutaneous sensory neurons,^40^ TRPA1 in trigeminal afferents,^41^ VEGF‐A in human microvascular endothelial cells,^42^ and TRIO and F‐actin binding protein (TRIOBP) and integrin, beta 5 (ITGβ5) in SMMC‐7721 cells.^43^ However, the transcription regulations by ARTN‐mediated signalling are incompletely defined and require further investigation.

## 4 THE ROLE OF ARTN IN SPINAL CORD INJURY REPAIR

Spinal cord injury (SCI) is debilitating and often has a poor prognosis, significantly due to our lack of understanding of its complex pathogenesis. Functional recovery following SCI requires axon regrowth into the site of injury with synaptic connectivity to target tissues.^44^ Increasing evidence shows that ARTN plays an important role in the functional recovery after spinal cord injury.^39^, ^45^, ^46^, ^47^ Further, ARTN was found to enhance neurite extension in vitro by overcoming myelin inhibition and may have the potential to improve motor recovery following SCI in a rodent model.^39^ Consistently, ARTN has been shown to preserve small types of nerve fibres, and type C fibre conduction velocity in the dorsal horn after SCI in a rodent model,^30^ and administration of ARTN led to the formation of sensory fibres reaching to the site of injury, and facilitated the restoration of sensory function following SCI.^48^ ARTN also modulates neurite initiation and neurite elongation, and the branching of unmyelinated sensory neurons in the spinal cord dorsal root ganglion (DRG) of mature rats,^29^, ^49^ as well as the regeneration of myelinated axons.^50^ Further, in DRG injury rats, the expressions of GFRα1 and GFRα3 were increased whereas the expression of GFRα2 was unchanged.^51^ ARTN was found to promote neurite outgrowth and actin polymerization in mature DRG by affecting the transcription of many target genes^49^ and stimulate the topographically correct regeneration of DRG axons in rodent dorsal root crush models.^52^ ARTN also induced and the regeneration of large, myelinated sensory afferents,^46^ and the peripheral nerve regeneration, and functional restoration of nerve fibres in partial lesions distal to DRG,^53^ which may be partially attributed to decreased caspase 3 and activating transcription factor 3 (ATF3) gene expression.^53^

## 5 THE ROLE OF ARTN IN NEUROPATHIC PAIN

ARTN and fellow GDNF family members also regulate the sensitivity of thermal nociceptors and hyperalgesia induced by inflammation,^54^ which appears to be mediated by ARTN‐GFRα3 interactions.^55^ Systemically administered ARTN was able to restore nociceptive and sensorimotor functions following injury.^48^ Further, peripherally derived ARTN appeared to play an important role in inflammatory and neuropathic pain through the regulation of TRPV1/A1 (members of the TRP family of cation channels, that are activated by noxious thermal and chemical stimuli) expression in primary afferent neurons in DRG.^56^ ARTN also modulates heat hyperalgesia and cold responses via TRPM8‐associated signalling in mice,^57^ which might involve the regulation of nicotinic acetylcholine receptor (nAChR) gene expression in thermal hypersensitivity.^58^ In the light of the important role of ARTN in neuropathic pain, ARTN might serve as a potential target for pain therapeutics.^59^ For example, small molecule BT13 that mimics the effect of ARTN has been shown to selectively activate RET signalling and to support neurite growth in a similar way to ARTN, providing additional indication of the potential of ARTN for developing medications to treat neuropathic pain.^60^ In particular, ARTN could be delivered selectively to neurons that are responsible for cold pain,^61^ potentially through modulation of the expression of TRPV1 (a polymodal calcium‐permeable cation channel activated by heat and inflammatory stimuli) and TRPA1 (an ionotropic channel responsive to cold and chemical stimuli) in cutaneous sensory neurons.^40^ Recently, ARTN (BG00010) was trialled clinically for pain relief of unilateral sciatica.^62^, ^63^ Results of the trial supported the further development of ARTN (BG00010) for the treatment of neuropathic pain.^62^ Further, randomized, double‐blinded, placebo‐controlled phase 2 trial showed evidence of pain relief ARTN (BG00010) with adverse event of headache, feeling hot and pruritus.^64^ Consistently, ARTN appears to mediate hypersensitivity and itch to warmth, leading to abnormal peripheral innervation, thermal hyperalgesia and provoked itch sensation in pruritic skin disorders, such as atopic dermatitis (AD).^65^ More recently, ARTN is involved in bone pain behaviour in a model of inflammatory bone via the activation and sensitization of bone afferent neurons.^66^

## 6 THE ROLE OF ARTN IN OTHER NEUROLOGICAL DISORDERS

In additional to SCI, increasingly data have implicated a role of ARTN in other neurological disorders. For instance, ARTN was found to induce neurite outgrowth from sympathetic neurons in the early stages of embryos^67^ and exert distinct effects on the generation, survival and growth of sympathetic neurones in vivo.^68^ ARTN could also enhance the regeneration of sensory axons in the brainstem.^46^ ARTN was detected expressed in blood vessels during periods of early sympathetic differentiation, where is it considered to be a guidance factor, possibly by chemoattractive activity, for the growth of sympathetic fibres.^11^ Using in vitro isolated mouse embryonic motor neurons, ARTN was shown to act as a survival factor for parasympathetic preganglionic motor neurons through GFRα3/Syndecan‐3 activation.^69^, ^70^ Further, ARTN was found to improve functional outcome after sciatic nerve injuries in rats.^70^ In that study, the sciatic nerve was transected and treated with a fibrin sealant containing ARTN, and the results revealed that ARTN increased the number of regenerating motor neurons.^70^ Interestingly, ARTN receptor GFRα3 was found expressed in Schwann cells and not in motor neurons, suggesting that the effect on motor neuron axon regeneration was due to a paracrine effect through Schwann cells in the injured nerve.^70^ Lentivirus‐based transfer of the ARTN gene demonstrated a neuroprotective effect on the nigral dopamine neurons in vivo, comparable to GDNF.^5^ It is generally accepted that dopamine neurons do not express GFRa3, and in the absence of GFRa3 from dopamine neurons, ARTN (in high concentration) is likely to act via GFRa1. Further, a GFRα3 knockout mice study revealed that GFRα3 signalling was required for the rostral migration of the superior cervical ganglion (SCG) precursors and for the survival of mature SCG neurons.^71^ GFLs, including ARTN, may provide a neuroprotective effect against excitotoxicity induced by compounds, such as *N*‐methyl‐d‐aspartate (NMDA), as determined in a culture model of hippocampal brain slices.^72^

In addition, gene polymorphisms or mutations of ARTN appear to be associated with Hirschsprung disease, a developmental disorder in the enteric nervous system.^73^, ^74^ ARTN is also implicated in the antidepressant activity of acetyl‐l‐carnitine (ALCAR),^75^ in dural afferent activity and migraine pain through modulation of primary afferent and sympathetic systems.^76^ Further, the expression of ARTN is reported to be associated with other neurological disorders, including generalized anxiety disorder (GAD),^77^ the pathogenesis of migraine,^78^ the iNOS‐mediated trigeminal pain pathway,^79^ a hereditary form of ptosis^80^ and autonomic neural dysplasia.^81^ Upregulation of ARTN mRNA was detected in the auditory nerve in association with deafness, indicating a possible role in the regulation of the auditory nerve system.^82^ Transgenic overexpression of ARTN in the tongue increases the expression of TRPV1 and TRPA1 in trigeminal afferents with altered oral sensation.^41^ ARTN also augments survival and axon regeneration in axotomized retinal ganglion cells in the optic nerve system.^83^

## 7 THE ROLE OF ARTN IN NON‐NEUROLOGICAL DISORDERS

In addition to neural tissues, ARTN might affect non‐neural organs. For instance, ARTN is a strong attractant of gut haematopoietic cells, inducing the formation of ectopic Peyer's patch‐like structures, which is suggestive of a role in intestine organogenesis.^84^ It was revealed that haematopoietic cells in the gut exhibit a random pattern of motility before forming the unique primordia structure of Peyer's patches via RET signalling. Knockout mice study showed that GFRa3 deficiency exhibited impaired Peyer's patch development, suggesting that ARTN/ GFRa3/RET mediates this process.^84^ Overexpression of ARTN in chronic pancreatitis disturbs tissue homoeostasis, leading to pancreatic fibrosis.^85^ Using quantitative PCR (polymerase chain reaction) and Western blot analyses, it was found that ARTN and GFRα3 were significantly overexpressed in chronic pancreatitis, and positively correlated with the severity of fibrosis. Further, transforming growth factor beta1 (TGF‐β1) upregulated the expression of ARTN in human pancreatic stellate cells (hPSCs) which might contribute to this pathogenesis.^85^

## 8 THE EMERGING ROLE OF ARTN IN CANCERS

Recently, a role of ARTN in tumorigenesis, tumour metastasis and drug resistance is emerging.^86^ ARTN was found to promote metastasis and poor survival outcome in patients with estrogen receptor (ER) negative mammary carcinoma (ER‐MC) via its cooperation with twist family BHLH transcription factor 1 (TWIST1). In this study, using a cohort of patients with ER‐MC and ER‐MC cell lines, it was revealed that overexpression of both ARTN and TWIST1 was associated with a poor survival outcome, whereas underexpression of both ARTN and TWIST1 predicted complete relapse free and overall survival in patients with ER‐MC. Further, in vitro assays showed that ARTN promoted an increase in TWIST1 expression via the activation of AKT/PKB (protein kinase B) pathway, and knockdown of TWIST1 expression by siRNA abolished ARTN‐mediated cellular metastasis behaviour.^87^ ARTN also plays a role in mammary carcinoma progression and metastasis via enhancing endothelial cell proliferation, migration, invasion and Matrigel tube formation. Using xenograft experiments, mammary carcinoma cells overexpressing ARTN were found to induce tumour formation with increased microvessel density, accompanied by increased VEGF‐A expression.^88^ ARTN expression induced by oestrogen in mammary carcinoma is involved in resistance to tamoxifen therapy, whereas antagonism of ARTN appears to enhance the efficacy of antioestrogens and may represent an adjunctive therapeutic approach.^89^ Genetic manipulation studies indicate that upregulation of ARTN increases the resistance of mammary carcinoma cells to trastuzumab; silencing of ARTN enhanced the efficacy of trastuzumab.^90^ ARTN was found to enhance the population of CSCs and increase the resistance of CSCs to trastuzumab via upregulation of BCL‐2 in vitro.^90^ The expression of ARTN and receptor subunits may predict the progression and outcome of mammary carcinoma subtypes.^91^

In non‐small‐cell lung carcinoma (NSCLC), ARTN was identified to arbitrate the progression of human NSCLC, as determined by clinical and laboratory findings.^92^ NSCLC tissues showed increased expression of ARTN and advanced lymph node metastasis, which was accompanied by increased migration and invasion of NSCLC cells via upregulation of BCL2 transcription.^92^ Overexpression of ARTN was also found to promote the proliferation and invasiveness of lung cancer cells in vitro.^93^ Similarly, the prognostic significance of ARTN expression in laryngeal squamous cell carcinoma (LSCC) may serve as predictors of LSCC progression and outcome in patients with LSCC.^94^

In pancreatic adenocarcinoma (PCa), ARTN appears to participate in the generation of pancreatic neuropathy^95^ and to stimulate the invasion and neurotrophic function of PCa in vivo and in vitro.^96^ Consistently, ARTN and CXC chemokine receptor 4 (CXCR4) were found to be overexpressed in pancreatic cancer tissues, and the migration and invasion of pancreatic cancer cells appears to be promoted by Akt and ERK 1/2/NF‐κB signalling and the stromal cell‐derived factor 1α (SDF‐1α)/CXCR4 axis.^97^

In hepatocellular carcinoma (HCC), ARTN was found to promote the growth and progression of HCC based on increased clinical tissue expression, increased tumour size, increased relapse and shorter survival time.^98^ Further laboratory investigation found that HCC cells overexpressing ARTN demonstrated reduced apoptosis, increased proliferation and EMT, and increased motility via hypoxia‐induced factor 1‐α (HIF‐1α) regulated AKT signalling, indicating that ARTN may function in a hypoxic environment and promote HCC.^98^

In endometrial carcinoma (EC), the expression levels of ARTN proteins were found to be positively correlated with the stage of EC and lymphatic metastasis.^99^ Additionally, ARTN is implicated as a pathogenic factor for certain acute myeloid leukaemia (AML) patients, which warrants further investigation.^100^ Activation of ARTN/GFRα3‐mediated RET signalling in AML cells requires further clinical investigation.^100^ Further, downstream RET‐mTORC1 signalling is found to promote AML cell growth through the suppression of autophagy and stabilization of leukaemia‐genic drivers, indicating the potential of RET as a therapeutic target in subgroups of AML patients.^100^

More recently, it was found that in the enlarged spleen of hosts bearing advanced tumours, erythroblast‐like cells are enriched and boost tumour progression via producing ARTN into the blood.^43^ Using hepatocellular carcinoma (HCC) tissues from in HCC patients, the protein levels of GFRa3 and phosphorylated RET were examined by immunohistochemistry (IHC) with automated cell acquisition. It was revealed that higher GFRa3 mRNA expression and RET phosphorylation in HCC tissues were correlated with the reduced disease‐free survival of HCC patients. Further, the higher levels of serum ARTN were correlated with the higher levels of GFRa3 expression or higher RET phosphorylation in HCC tissues in patients with shorter disease‐free survival.^43^ Consistently, in vivo depletion or deficiency of ARTN inhibits the growth of HCC and abolishes tumour‐promoting ability of erythroblast‐like cells.^43^

**FIGURE 4:**
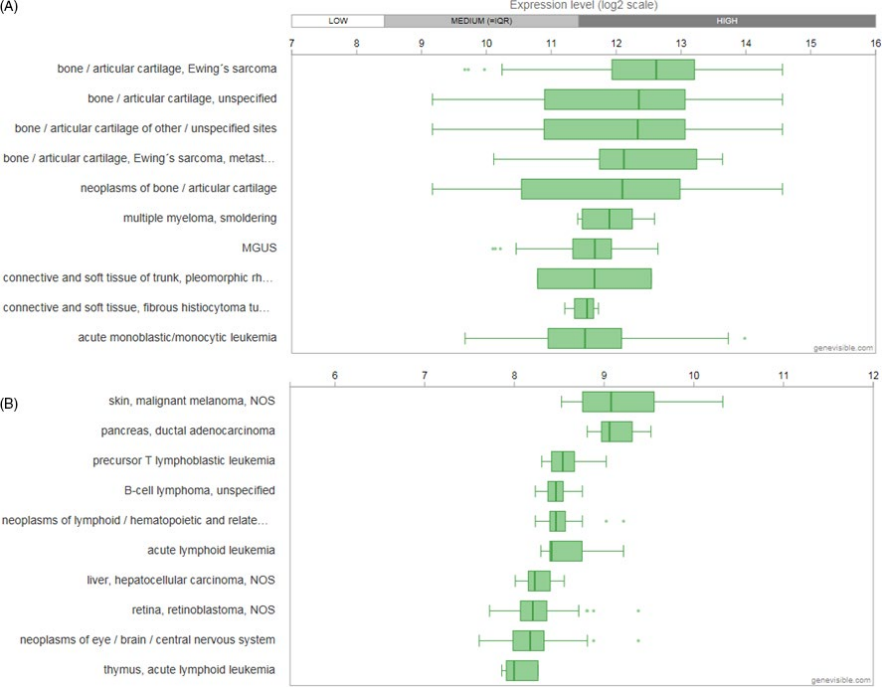
Bioinformatics analyses of ARTN gene expression in cancers. A, 10 human cancer types containing highest ARTN expression levels. B, 10 mouse cancer types containing highest ARTN expression levels. Analyses were performed by Genevisible^®^ bioinformatics based on data from Affymetrix human genome U133 plus 2.0 array, and Affymetrix mouse genome 430 2.0 array, respectively (http://genevisible.com)

Bioinformatics gene analyses indicate that the ARTN transcripts are expressed in human and mouse cancers, most abundantly in Ewing's sarcoma in human and in melanoma in mouse (Figure 4).^101^ However, the mechanism by which ARTN is differentially expressed and involved in various types of cancers warrants further investigation.

## 9 CONCLUSIONS

ARTN, a member of GDNF family ligands, plays a fundamental role in the development and homoeostasis of the nervous system. ARTN is conserved among mammalian species and bears characteristics of the TGF‐β superfamily. Mechanistically, ARTN interacts preferentially with receptor GFRα3, forming a signalling complex with RET which induces intracellular pathways and the transcriptional activity of target genes. Evidence suggests that ARTN plays an important role in functional recovery and neural regeneration following SCI and peripheral nerve injury. ARTN appears to be involved in other neurological disorders, such as Hirschsprung and motor neuron disease. ARTN also attracts hematopoietic cells to participate in the formation of Peyer's patch‐like lymphoid tissue in the gut. Emerging evidence indicates that ARTN is involved in the tumorigenesis, tumour metastasis and therapeutic resistance to cancers, such as mammary carcinoma, pancreatic adenocarcinoma and acute myeloid leukaemia. ARTN, therefore, emerges as a potential therapeutic target for regenerative medical applications in the treatment of spinal cord injury, neuropathic pain and cancers. Further research is needed to develop the therapeutic potential of ARTN and GFL members, including GDNF.

## CONFLICT OF INTEREST

No conflict of interest.

## AUTHOR CONTRIBUTION

Sipin Zhu and Yihe Li conducted research and drafted the manuscript. Samuel Bennett, Junhao Chen, Isabel Ziwai Weng and Lin Huang performed the protein structural analysis and provided evaluation and assistance in the process of drafting and revision of the manuscript. Huazi Xu and Jiake Xu supervised the study and revised the manuscript.

## Data Availability

The data that support the findings of this study are available from the corresponding author upon reasonable request.

## References

[1] Lin LF , Doherty DH , Lile JD , Bektesh S , Collins F . GDNF: a glial cell line‐derived neurotrophic factor for midbrain dopaminergic neurons. Science. 1993;260(5111):1130‐1132. 8493557 10.1126/science.8493557

[2] Airaksinen MS , Saarma M . The GDNF family: signalling, biological functions and therapeutic value. Nat Rev Neurosci. 2002;3(5):383‐394. 11988777 10.1038/nrn812

[3] Kotzbauer PT , Lampe PA , Heuckeroth RO , et al. Neurturin, a relative of glial‐cell‐line‐derived neurotrophic factor. Nature. 1996;384(6608):467‐470. 8945474 10.1038/384467a0

[4] Masure S , Geerts H , Cik M , et al. Enovin, a member of the glial cell‐line‐derived neurotrophic factor (GDNF) family with growth promoting activity on neuronal cells. Existence and tissue‐specific expression of different splice variants. Eur J Biochem. 1999;266(3):892‐902. 10583383 10.1046/j.1432-1327.1999.00925.x

[5] Rosenblad C , Gronborg M , Hansen C , et al. In vivo protection of nigral dopamine neurons by lentiviral gene transfer of the novel GDNF‐family member neublastin/artemin. Mol Cell Neurosci. 2000;15(2):199‐214. 10673327 10.1006/mcne.1999.0817

[6] Saarma M . GDNF – a stranger in the TGF‐beta superfamily? Eur J Biochem. 2000;267(24):6968‐6971. 11106404 10.1046/j.1432-1327.2000.01826.x

[7] Sariola H , Saarma M . Novel functions and signalling pathways for GDNF. J Cell Sci. 2003;116(Pt 19):3855‐3862. 12953054 10.1242/jcs.00786

[8] Chang H , Brown CW , Matzuk MM . Genetic analysis of the mammalian transforming growth factor‐beta superfamily. Endocr Rev. 2002;23(6):787‐823. 12466190 10.1210/er.2002-0003

[9] Baloh RH , Tansey MG , Lampe PA , et al. Artemin, a novel member of the GDNF ligand family, supports peripheral and central neurons and signals through the GFRalpha3‐RET receptor complex. Neuron. 1998;21(6):1291‐1302. 9883723 10.1016/s0896-6273(00)80649-2

[10] Zihlmann KB , Ducray AD , Schaller B , et al. The GDNF family members neurturin, artemin and persephin promote the morphological differentiation of cultured ventral mesencephalic dopaminergic neurons. Brain Res Bull. 2005;68(1‐2):42‐53. 16325003 10.1016/j.brainresbull.2004.10.012

[11] Honma Y , Araki T , Gianino S , et al. Artemin is a vascular‐derived neurotropic factor for developing sympathetic neurons. Neuron. 2002;35(2):267‐282. 12160745 10.1016/s0896-6273(02)00774-2

[12] Gardell LR , Wang R , Ehrenfels C , et al. Multiple actions of systemic artemin in experimental neuropathy. Nat Med. 2003;9(11):1383‐1389. 14528299 10.1038/nm944

[13] Bruinzeel W , Masure S . Exceptional stability of artemin neurotrophic factor dimers: effects of temperature, pH, buffer and storage conditions on protein integrity and activity. Appl Biochem Biotechnol. 2011;165(5‐6):1379‐1390. 21892665 10.1007/s12010-011-9354-4

[14] Bruinzeel W , Masure S . Recombinant expression, purification and dimerization of the neurotrophic growth factor Artemin for in vitro and in vivo use. Protein Expr Purif. 2012;81(1):25‐32. 21907286 10.1016/j.pep.2011.08.028

[15] Silvian L , Jin P , Carmillo P , et al. Artemin crystal structure reveals insights into heparan sulfate binding. Biochemistry. 2006;45(22):6801‐6812. 16734417 10.1021/bi060035x

[16] Fontana X , Hristova M , Da Costa C , et al. c‐Jun in Schwann cells promotes axonal regeneration and motoneuron survival via paracrine signaling. J Cell Biol. 2012;198(1):127‐141. 22753894 10.1083/jcb.201205025 PMC3392945

[17] Lam PK , Wang KKW , Lo AWI , et al. Interactome and reciprocal activation of pathways in topical mesenchymal stem cells and the recipient cerebral cortex following traumatic brain injury. Sci Rep. 2017;7(1):5017. 28694468 10.1038/s41598-017-01772-7 PMC5504061

[18] Treanor JJ , Goodman L , de Sauvage F , et al. Characterization of a multicomponent receptor for GDNF. Nature. 1996;382(6586):80‐83. 8657309 10.1038/382080a0

[19] Durbec P , Marcos‐Gutierrez CV , Kilkenny C , et al. GDNF signalling through the Ret receptor tyrosine kinase. Nature. 1996;381(6585):789‐793. 8657282 10.1038/381789a0

[20] Worby CA , Vega QC , Zhao Y , Chao HH , Seasholtz AF , Dixon JE . Glial cell line‐derived neurotrophic factor signals through the RET receptor and activates mitogen‐activated protein kinase. J Biol Chem. 1996;271(39):23619‐23622. 8798576 10.1074/jbc.271.39.23619

[21] Airaksinen MS , Titievsky A , Saarma M . GDNF family neurotrophic factor signaling: four masters, one servant? Mol Cell Neurosci. 1999;13(5):313‐325. 10356294 10.1006/mcne.1999.0754

[22] Wang X , Baloh RH , Milbrandt J , Garcia KC . Structure of artemin complexed with its receptor GFRalpha3: convergent recognition of glial cell line‐derived neurotrophic factors. Structure. 2006;14(6):1083‐1092. 16765900 10.1016/j.str.2006.05.010

[23] Enomoto H , Crawford PA , Gorodinsky A , Heuckeroth RO , Johnson EM Jr , Milbrandt J . RET signaling is essential for migration, axonal growth and axon guidance of developing sympathetic neurons. Development. 2001;128(20):3963‐3974. 11641220 10.1242/dev.128.20.3963

[24] Widenfalk J , Widmer HR , Spenger C . GDNF, RET and GFRalpha‐1‐3 mRNA expression in the developing human spinal cord and ganglia. NeuroReport. 1999;10(7):1433‐1439. 10380959 10.1097/00001756-199905140-00009

[25] Naveilhan P , Baudet C , Mikaels A , Shen L , Westphal H , Ernfors P . Expression and regulation of GFRalpha3, a glial cell line‐derived neurotrophic factor family receptor. Proc Natl Acad Sci USA. 1998;95(3):1295‐1300. 9448325 10.1073/pnas.95.3.1295 PMC18749

[26] Shin E , Hong SW , Kim SH , Yang WI . Expression of down stream molecules of RET (p‐ERK, p‐p38 MAPK, p‐JNK and p‐AKT) in papillary thyroid carcinomas. Yonsei Med J. 2004;45(2):306‐313. 15119004 10.3349/ymj.2004.45.2.306

[27] Ichihara M , Murakumo Y , Takahashi M . RET and neuroendocrine tumors. Cancer Lett. 2004;204(2):197‐211. 15013219 10.1016/S0304-3835(03)00456-7

[28] Jeong DG , Park WK , Park S . Artemin activates axonal growth via SFK and ERK‐dependent signalling pathways in mature dorsal root ganglia neurons. Cell Biochem Funct. 2008;26(2):210‐220. 17868192 10.1002/cbf.1436

[29] Wong AW , Yeung JKP , Payne SC , Keast JR , Osborne PB . Neurite outgrowth in normal and injured primary sensory neurons reveals different regulation by nerve growth factor (NGF) and artemin. Mol Cell Neurosci. 2015;65:125‐134. 25752731 10.1016/j.mcn.2015.03.004

[30] Bennett DL , Boucher TJ , Michael GJ , et al. Artemin has potent neurotrophic actions on injured C‐fibres. J Peripher Nerv Syst. 2006;11(4):330‐345. 17117942 10.1111/j.1529-8027.2006.00106.x

[31] Saarma M , Sariola H . Other neurotrophic factors: glial cell line‐derived neurotrophic factor (GDNF). Microsc Res Tech. 1999;45(4–5):292‐302. 10383122 10.1002/(SICI)1097-0029(19990515/01)45:4/5<292::AID-JEMT13>3.0.CO;2-8

[32] Paratcha G , Ledda F , Ibanez CF . The neural cell adhesion molecule NCAM is an alternative signaling receptor for GDNF family ligands. Cell. 2003;113(7):867‐879. 12837245 10.1016/s0092-8674(03)00435-5

[33] Carmillo P , Dago L , Day ES , et al. Glial cell line‐derived neurotrophic factor (GDNF) receptor alpha‐1 (GFR alpha 1) is highly selective for GDNF versus artemin. Biochemistry. 2005;44(7):2545‐2554. 15709767 10.1021/bi049247p

[34] Rakowicz WP , Staples CS , Milbrandt J , Brunstrom JE , Johnson EM Jr . Glial cell line‐derived neurotrophic factor promotes the survival of early postnatal spinal motor neurons in the lateral and medial motor columns in slice culture. J Neurosci. 2002;22(10):3953‐3962. 12019314 10.1523/JNEUROSCI.22-10-03953.2002 PMC6757646

[35] Ilieva M , Nielsen J , Korshunova I , et al. Artemin and an artemin‐derived peptide, artefin, induce neuronal survival, and differentiation through ret and NCAM. Front Mol Neurosci. 2019;12:47. 30853893 10.3389/fnmol.2019.00047 PMC6396024

[36] Bespalov MM , Sidorova YA , Tumova S , et al. Heparan sulfate proteoglycan syndecan‐3 is a novel receptor for GDNF, neurturin, and artemin. J Cell Biol. 2011;192(1):153‐169. 21200028 10.1083/jcb.201009136 PMC3019558

[37] Fukuda T , Kiuchi K , Takahashi M . Novel mechanism of regulation of Rac activity and lamellipodia formation by RET tyrosine kinase. J Biol Chem. 2002;277(21):19114‐19121. 11886862 10.1074/jbc.M200643200

[38] Tee JB , Choi Y , Shah MM , et al. Protein kinase A regulates GDNF/RET‐dependent but not GDNF/Ret‐independent ureteric bud outgrowth from the Wolffian duct. Dev Biol. 2010;347(2):337‐347. 20816800 10.1016/j.ydbio.2010.08.029 PMC2981800

[39] Zhou Z , Peng X , Fink DJ , Mata M . HSV‐mediated transfer of artemin overcomes myelin inhibition to improve outcome after spinal cord injury. Mol Ther. 2009;17(7):1173‐1179. 19293775 10.1038/mt.2009.52 PMC2835217

[40] Elitt CM , McIlwrath SL , Lawson JJ , et al. Artemin overexpression in skin enhances expression of TRPV1 and TRPA1 in cutaneous sensory neurons and leads to behavioral sensitivity to heat and cold. J Neurosci. 2006;26(33):8578‐8587. 16914684 10.1523/JNEUROSCI.2185-06.2006 PMC6674358

[41] Elitt CM , Malin SA , Koerber HR , Davis BM , Albers KM . Overexpression of artemin in the tongue increases expression of TRPV1 and TRPA1 in trigeminal afferents and causes oral sensitivity to capsaicin and mustard oil. Brain Res. 2008;1230:80‐90. 18652806 10.1016/j.brainres.2008.06.119 PMC2570744

[42] Banerjee A , Wu ZS , Qian PX , et al. ARTEMIN promotes de novo angiogenesis in ER negative mammary carcinoma through activation of TWIST1‐VEGF‐A signalling. PLoS One. 2012;7(11):e50098. 23185544 10.1371/journal.pone.0050098 PMC3503764

[43] Han Y , Liu Q , Hou J , et al. Tumor‐induced generation of splenic erythroblast‐like Ter‐cells promotes tumor progression. Cell. 2018;173(3):634–648.e12. 29606356 10.1016/j.cell.2018.02.061

[44] Abrams M , Widenfalk J . Emerging strategies to promote improved functional outcome after peripheral nerve injury. Restor Neurol Neurosci. 2005;23(5‐6):367‐382. 16477099

[45] Piltti KM , Funes GM , Avakian SN , et al. Increasing human neural stem cell transplantation dose alters oligodendroglial and neuronal differentiation after spinal cord injury. Stem Cell Rep. 2017;8(6):1534‐1548. 10.1016/j.stemcr.2017.04.009 PMC5469937 28479305

[46] Wong LE , Gibson ME , Arnold HM , Pepinsky B , Frank E . Artemin promotes functional long‐distance axonal regeneration to the brainstem after dorsal root crush. Proc Natl Acad Sci USA. 2015;112(19):6170‐6175. 25918373 10.1073/pnas.1502057112 PMC4434726

[47] Detloff MR , Smith EJ , Quiros Molina D , Ganzer PD , Houle JD . Acute exercise prevents the development of neuropathic pain and the sprouting of non‐peptidergic (GDNF‐ and artemin‐responsive) c‐fibers after spinal cord injury. Exp Neurol. 2014;255:38‐48. 24560714 10.1016/j.expneurol.2014.02.013 PMC4036591

[48] Wang R , King T , Ossipov MH , et al. Persistent restoration of sensory function by immediate or delayed systemic artemin after dorsal root injury. Nat Neurosci. 2008;11(4):488‐496. 18344995 10.1038/nn2069 PMC3417340

[49] Park S , Hong YW . Transcriptional regulation of artemin is related to neurite outgrowth and actin polymerization in mature DRG neurons. Neurosci Lett. 2006;404(1–2):61‐66. 16781061 10.1016/j.neulet.2006.05.041

[50] Kelamangalath L , Tang X , Bezik K , Sterling N , Son YJ , Smith GM . Neurotrophin selectivity in organizing topographic regeneration of nociceptive afferents. Exp Neurol. 2015;271:262‐278. 26054884 10.1016/j.expneurol.2015.06.007 PMC5090981

[51] Keast JR , Forrest SL , Osborne PB . Sciatic nerve injury in adult rats causes distinct changes in the central projections of sensory neurons expressing different glial cell line‐derived neurotrophic factor family receptors. J Comp Neurol. 2010;518(15):3024‐3045. 20533358 10.1002/cne.22378 PMC2883785

[52] Harvey P , Gong B , Rossomando AJ , Frank E . Topographically specific regeneration of sensory axons in the spinal cord. Proc Natl Acad Sci USA. 2010;107(25):11585‐11590. 20534446 10.1073/pnas.1003287107 PMC2895079

[53] Wang R , Rossomando A , Sah DW , Ossipov MH , King T , Porreca F . Artemin induced functional recovery and reinnervation after partial nerve injury. Pain. 2014;155(3):476‐484. 24269493 10.1016/j.pain.2013.11.007 PMC3936608

[54] Malin SA , Molliver DC , Koerber HR , et al. Glial cell line‐derived neurotrophic factor family members sensitize nociceptors in vitro and produce thermal hyperalgesia in vivo. J Neurosci. 2006;26(33):8588‐8599. 16914685 10.1523/JNEUROSCI.1726-06.2006 PMC6674355

[55] Thornton P , Hatcher JP , Robinson I , et al. Artemin‐GFRalpha3 interactions partially contribute to acute inflammatory hypersensitivity. Neurosci Lett. 2013;545:23‐28. 23603259 10.1016/j.neulet.2013.04.007

[56] Ikeda‐Miyagawa Y , Kobayashi K , Yamanaka H , et al. Peripherally increased artemin is a key regulator of TRPA1/V1 expression in primary afferent neurons. Mol Pain. 2015;11:8. 25889103 10.1186/s12990-015-0004-7 PMC4357199

[57] Lippoldt EK , Elmes RR , McCoy DD , Knowlton WM , McKemy DD . Artemin, a glial cell line‐derived neurotrophic factor family member, induces TRPM8‐dependent cold pain. J Neurosci. 2013;33(30):12543‐12552. 23884957 10.1523/JNEUROSCI.5765-12.2013 PMC3721853

[58] Albers KM , Zhang XL , Diges CM , et al. Artemin growth factor increases nicotinic cholinergic receptor subunit expression and activity in nociceptive sensory neurons. Mol Pain. 2014;10:31. 24886596 10.1186/1744-8069-10-31 PMC4036648

[59] Sah DW , Ossipov MH , Rossomando A , Silvian L , Porreca F . New approaches for the treatment of pain: the GDNF family of neurotrophic growth factors. Curr Top Med Chem. 2005;5(6):577‐583. 16022680 10.2174/1568026054367593

[60] Sidorova YA , Bespalov MM , Wong AW , et al. A novel small molecule GDNF receptor RET agonist, BT13, promotes neurite growth from sensory neurons in vitro and attenuates experimental neuropathy in the rat. Front Pharmacol. 2017;8:365. 28680400 10.3389/fphar.2017.00365 PMC5478727

[61] Lippoldt EK , Ongun S , Kusaka GK , McKemy DD . Inflammatory and neuropathic cold allodynia are selectively mediated by the neurotrophic factor receptor GFRalpha3. Proc Natl Acad Sci USA. 2016;113(16):4506‐4511. 27051069 10.1073/pnas.1603294113 PMC4843462

[62] Rolan PE , O'Neill G , Versage E , et al. First‐in‐human, double‐blind, placebo‐controlled, randomized, Dose‐Escalation Study of BG00010, a Glial cell line‐derived neurotrophic factor family member, in subjects with unilateral sciatica. PLoS One. 2015;10(5):e0125034. 25962165 10.1371/journal.pone.0125034 PMC4427304

[63] Okkerse P , Hay JL , Versage E , et al. Pharmacokinetics and pharmacodynamics of multiple doses of BG00010, a neurotrophic factor with anti‐hyperalgesic effects, in patients with sciatica. Br J Clin Pharmacol. 2016;82(1):108‐117. 27016000 10.1111/bcp.12941 PMC4917813

[64] Backonja M , Williams L , Miao X , Katz N , Chen C . Safety and efficacy of neublastin in painful lumbosacral radiculopathy: a randomized, double‐blinded, placebo‐controlled phase 2 trial using Bayesian adaptive design (the SPRINT trial). Pain. 2017;158(9):1802‐1812. 28746076 10.1097/j.pain.0000000000000983 PMC5761750

[65] Murota H , Izumi M , Abd El‐Latif MI , et al. Artemin causes hypersensitivity to warm sensation, mimicking warmth‐provoked pruritus in atopic dermatitis. J Allergy Clin Immunol. 2012;130(3):671‐682.e4. 22770266 10.1016/j.jaci.2012.05.027

[66] Nencini S , Ringuet M , Kim DH , Greenhill C , Ivanusic JJ . GDNF, Neurturin, and artemin activate and sensitize bone afferent neurons and contribute to inflammatory bone pain. J Neurosci. 2018;38(21):4899‐4911. 29712778 10.1523/JNEUROSCI.0421-18.2018 PMC6596122

[67] Yan H , Newgreen DF , Young HM . Developmental changes in neurite outgrowth responses of dorsal root and sympathetic ganglia to GDNF, neurturin, and artemin. Dev Dyn. 2003;227(3):395‐401. 12815625 10.1002/dvdy.10294

[68] Andres R , Forgie A , Wyatt S , Chen Q , de Sauvage FJ , Davies AM . Multiple effects of artemin on sympathetic neurone generation, survival and growth. Development. 2001;128(19):3685‐3695. 11585795 10.1242/dev.128.19.3685

[69] Schaller S , Buttigieg D , Alory A , et al. Novel combinatorial screening identifies neurotrophic factors for selective classes of motor neurons. Proc Natl Acad Sci USA. 2017;114(12):E2486‐E2493. 28270618 10.1073/pnas.1615372114 PMC5373341

[70] Widenfalk J , Wu W , Hao J , Person JK , Wiesenfeldt‐Hallin Z , Risling M . Treatment of transected peripheral nerves with artemin improved motor neuron regeneration, but did not reduce nerve injury‐induced pain behaviour. Scand J Plast Reconstr Surg Hand Surg. 2009;43(5):245‐250. 19863426 10.3109/02844310903259082

[71] Nishino J , Mochida K , Ohfuji Y , et al. GFR alpha3, a component of the artemin receptor, is required for migration and survival of the superior cervical ganglion. Neuron. 1999;23(4):725‐736. 10482239 10.1016/s0896-6273(01)80031-3

[72] Bonde C , Kristensen BW , Blaabjerg M , Johansen TE , Zimmer J , Meyer M . GDNF and neublastin protect against NMDA‐induced excitotoxicity in hippocampal slice cultures. NeuroReport. 2000;11(18):4069‐4073. 11192630 10.1097/00001756-200012180-00032

[73] Fernandez RM , Ruiz‐Ferrer M , Lopez‐Alonso M , Antinolo G , Borrego S . Polymorphisms in the genes encoding the 4 RET ligands, GDNF, NTN, ARTN, PSPN, and susceptibility to Hirschsprung disease. J Pediatr Surg. 2008;43(11):2042‐2047. 18970938 10.1016/j.jpedsurg.2008.05.018

[74] Ruiz‐Ferrer M , Torroglosa A , Luzon‐Toro B , et al. Novel mutations at RET ligand genes preventing receptor activation are associated to Hirschsprung's disease. J Mol Med (Berl). 2011;89(5):471‐480. 21206993 10.1007/s00109-010-0714-2

[75] Di Cesare ML , Vivoli E , Salvicchi A , et al. Antidepressant‐like effect of artemin in mice: a mechanism for acetyl‐L‐carnitine activity on depression. Psychopharmacology. 2011;218(2):347‐356. 21590285 10.1007/s00213-011-2326-0

[76] McIlvried LA , Albers K , Gold MS . Distribution of artemin and GFRalpha3 labeled nerve fibers in the dura mater of rat: artemin and GFRalpha3 in the dura. Headache. 2010;50(3):442‐450. 19845789 10.1111/j.1526-4610.2009.01548.x PMC3074600

[77] Pallanti S , Tofani T , Zanardelli M , Di Cesare ML , Ghelardini C . BDNF and Artemin are increased in drug‐naive non‐depressed GAD patients: preliminary data. Int J Psychiatry Clin Pract. 2014;18(4):255‐260. 24994477 10.3109/13651501.2014.940051

[78] Shang HQ , Wang Y , Mao YY , et al. Expression of artemin and GFRalpha3 in an animal model of migraine: possible role in the pathogenesis of this disorder. J Headache Pain. 2016;17(1):81. 27600145 10.1186/s10194-016-0673-2 PMC5013005

[79] Shang H , Wang Y , Chao X , et al. Artemin transiently increases iNOS expression in primary cultured trigeminal ganglion neurons. Neurosci Lett. 2017;660:34‐38. 28899786 10.1016/j.neulet.2017.09.016

[80] Chen CK , Mungall CJ , Gkoutos GV , et al. MouseFinder: Candidate disease genes from mouse phenotype data. Hum Mutat. 2012;33(5):858‐866. 22331800 10.1002/humu.22051 PMC3327758

[81] Bolon B , Jing S , Asuncion F , et al. The candidate neuroprotective agent artemin induces autonomic neural dysplasia without preventing peripheral nerve dysfunction. Toxicol Pathol. 2004;32(3):275‐294. 15204970 10.1080/01926230490431475

[82] Wissel K , Wefstaedt P , Rieger H , Miller JM , Lenarz T , Stover T . Upregulation of glial cell line‐derived neurotrophic factor and artemin mRNA in the auditory nerve of deafened rats. NeuroReport. 2006;17(9):875‐878. 16738479 10.1097/01.wnr.0000221836.26093.85

[83] Omodaka K , Kurimoto T , Nakamura O , et al. Artemin augments survival and axon regeneration in axotomized retinal ganglion cells. J Neurosci Res. 2014;92(12):1637‐1646. 25044131 10.1002/jnr.23449

[84] Veiga‐Fernandes H , Coles MC , Foster KE , et al. Tyrosine kinase receptor RET is a key regulator of Peyer's patch organogenesis. Nature. 2007;446(7135):547‐551. 17322904 10.1038/nature05597

[85] Ceyhan GO , Bergmann F , Kadihasanoglu M , et al. The neurotrophic factor artemin influences the extent of neural damage and growth in chronic pancreatitis. Gut. 2007;56(4):534‐544. 17047099 10.1136/gut.2006.105528 PMC1856869

[86] Hezam K , Jiang J , Sun F , Zhang X , Zhang J . Artemin promotes oncogenicity, metastasis and drug resistance in cancer cells. Rev Neurosci. 2018;29(1):93‐98. 28937965 10.1515/revneuro-2017-0029

[87] Banerjee A , Wu ZS , Qian P , et al. ARTEMIN synergizes with TWIST1 to promote metastasis and poor survival outcome in patients with ER negative mammary carcinoma. Breast Cancer Res. 2011;13(6):R112. 22060274 10.1186/bcr3054 PMC3326554

[88] Banerjee A , Qian P , Wu ZS , et al. Artemin stimulates radio‐ and chemo‐resistance by promoting TWIST1‐BCL‐2‐dependent cancer stem cell‐like behavior in mammary carcinoma cells. Biol Chem. 2012;287(51):42502‐42515. 10.1074/jbc.M112.365163 PMC3522252 23095743

[89] Kang J , Qian PX , Pandey V , et al. Artemin is estrogen regulated and mediates antiestrogen resistance in mammary carcinoma. Oncogene. 2010;29(22):3228‐3240. 20305694 10.1038/onc.2010.71

[90] Ding K , Banerjee A , Tan S , et al. Artemin, a member of the glial cell line‐derived neurotrophic factor family of ligands, is HER2‐regulated and mediates acquired trastuzumab resistance by promoting cancer stem cell‐like behavior in mammary carcinoma cells. J Biol Chem. 2014;289(23):16057‐16071. 24737320 10.1074/jbc.M113.529552 PMC4047380

[91] Wu ZS , Pandey V , Wu WY , Ye S , Zhu T , Lobie PE . Prognostic significance of the expression of GFRalpha1, GFRalpha3 and syndecan‐3, proteins binding ARTEMIN, in mammary carcinoma. BMC Cancer. 2013;13:34. 23351331 10.1186/1471-2407-13-34 PMC3562211

[92] Tang JZ , Kong XJ , Kang J , et al. Artemin‐stimulated progression of human non‐small cell lung carcinoma is mediated by BCL2. Mol Cancer Ther. 2010;9(6):1697‐1708. 20530713 10.1158/1535-7163.MCT-09-1077

[93] Song Z , Yang F , Du H , et al. Role of artemin in non‐small cell lung cancer. Thorac Cancer. 2018;9(5):555‐562. 29575549 10.1111/1759-7714.12615 PMC5928368

[94] Gao C , Cheng X , Li X , Tong B , Wu K , Liu Y . Prognostic significance of artemin and GFRalpha1 expression in laryngeal squamous cell carcinoma. Exp Ther Med. 2014;8(3):818‐822. 25120606 10.3892/etm.2014.1821 PMC4113528

[95] Ceyhan GO , Schafer KH , Kerscher AG , et al. Nerve growth factor and artemin are paracrine mediators of pancreatic neuropathy in pancreatic adenocarcinoma. Ann Surg. 2010;251(5):923‐931. 20395845 10.1097/SLA.0b013e3181d974d4

[96] Gao L , Bo H , Wang Y , Zhang J , Zhu M . Neurotrophic factor artemin promotes invasiveness and neurotrophic function of pancreatic adenocarcinoma in vivo and in vitro. Pancreas. 2015;44(1):134‐143. 25243385 10.1097/MPA.0000000000000223 PMC4272229

[97] Wang J , Wang H , Cai J , et al. Artemin regulates CXCR4 expression to induce migration and invasion in pancreatic cancer cells through activation of NF‐kappaB signaling. Exp Cell Res. 2018;365(1):12‐23. 29453972 10.1016/j.yexcr.2018.02.008

[98] Zhang M , Zhang W , Wu Z , et al. Artemin is hypoxia responsive and promotes oncogenicity and increased tumor initiating capacity in hepatocellular carcinoma. Oncotarget. 2016;7(3):3267‐3282. 26675549 10.18632/oncotarget.6572 PMC4823105

[99] Wang XH , Liu YN , Tian K , et al. Expression and clinical significance of ARTN and MMP‐9 in endometrial carcinoma. J Biol Regul Homeost Agents. 2017;31(4):879‐887. 29254290

[100] Rudat S , Pfaus A , Cheng YY , et al. RET‐mediated autophagy suppression as targetable co‐dependence in acute myeloid leukemia. Leukemia. 2018;32(10):2189‐2202. 29654265 10.1038/s41375-018-0102-4

[101] Hruz T , Laule O , Szabo G , et al. Genevestigator v3: a reference expression database for the meta‐analysis of transcriptomes. Adv Bioinformatics. 2008;2008:420747. 19956698 10.1155/2008/420747 PMC2777001

